# Bonobos modify communication signals according to recipient familiarity

**DOI:** 10.1038/srep16442

**Published:** 2015-11-10

**Authors:** Emilie Genty, Christof Neumann, Klaus Zuberbühler

**Affiliations:** 1 Department of Comparative Cognition, Institute of Biology, University of Neuchâtel, rue Emile Argand 11, 2000 Neuchâtel, Switzerland; 2 School of Psychology and Neuroscience, University of St Andrews, St Andrews, KY16 9JP, Scotland (UK)

## Abstract

Human and nonhuman primate communication differs in various ways. In particular, humans base communicative efforts on mutual knowledge and conventions shared between interlocutors. In this study, we experimentally tested whether bonobos (*Pan paniscus*), a close relative to humans, are able to take into account the familiarity, i.e. the shared interaction history, when communicating with a human partner. In five experimental conditions we found that subjects took the recipients’ attentional state and their own communicative effectiveness into account by adjusting signal production accordingly. More importantly, in case of communicative failure, subjects repeated previously successful signals more often with a familiar than unfamiliar recipient, with whom they had no previous interactions, and elaborated by switching to new signals more with the unfamiliar than the familiar one, similar to what has previously been found in two year-old children. We discuss these findings in relation to the human capacity to establish common ground between interlocutors, a crucial aspect of human cooperative communication.

Human communication is punctuated by misunderstandings. Recipients can resolve them by requesting clarifications from signallers, who then must discern why the recipient has failed to comprehend them. Problems with perception or unfamiliarity with a linguistic convention are common causes, which are detected by assessments of the recipient’s mental states. In child development, the transition to understanding others as mental agents is a gradual process that derives, at least partly, from understanding intentions^1^. This is particularly important in linguistic discourse in which there is a continued need to take the listener’s perspective into account, which often differs from the signaller’s own. In case of failure, listeners will signal miscomprehension, an ability already observed in two-years olds^2^,^3^,^4^,^5^. For example, misunderstood infants are more likely to elaborate linguistic requests to an unfamiliar than a familiar adult, indicating that they possess some understanding of their shared interaction history^6^,^7^. In humans, in other words, shared experiences between a signaller and a recipient determine what inferences can be made about mutual knowledge, a foundation necessary to communicate cooperatively^8^.

Human cognitive evolution is often investigated with comparative research on non-human primates and particularly great apes, which continues to contribute to a clearer understanding of how hominids have diverged from their primate ancestors and what features continue to be shared with modern apes. Particularly impressive are studies with ‘enculturated’ great apes that have acquired artificial linguistic systems to communicate with their human caretakers^9^,^10^. Results of these studies have regularly been interpreted in terms of language-relevant capacities (e.g.^11^,^12^,^13^), including the claim that bonobos are capable of taking into account shared knowledge when interacting with familiar humans^14^,^15^. A general conclusion from this literature is that key cognitive abilities necessary for language were already present in the common ancestor of modern humans, chimpanzees and bonobos.

Although impressive, one weakness of this body of research has always been the difficulty to find comparable capacities in the natural communication of wild apes (e.g.^16^,^17^,^18^). In recent years, however, there have been concerted efforts to systematically investigate whether great apes and other animals share core abilities required for language, such as intentionality, reference, mental concepts, vocal imitation or signal combinations^19^,^20^,^21^,^22^,^23^,^24^. For great apes, there is now little doubt that individuals can address others intentionally, usually to alter a recipient’s behaviour in a goal-directed way^25^,^26^,^27^,^28^,^29^ or to convey information referentially^30^,^31^. Primates also seem to understand the communicative function of some of their signals, in that they require the presence and attention of a suitable recipient, as documented by a large number of studies (e.g.^25^,^26^,^32^,^33^,^34^,^35^,^36^,^37^,^38^,^39^). Here, a standard experimental paradigm has been to analyse signal production when requesting food from a human experimenter whose attentional state, comprehension or intention varied. A key finding has been that both monkeys and apes are more likely to use visual signals when the experimenter is oriented towards them, and audible signals when the experimenter is facing away (e.g., apes: 33,39,40; monkeys:^41^,^42^,^43^,^44^,^45^). Another finding has been that chimpanzees and orang-utans adjust their signalling behaviour according to the degree of comprehension manifest in a partner. When confronted with a response that suggested only partial understanding, apes persisted and continued to use the signals that they used before. When fully misunderstood, they elaborated their communication efforts by producing new signals^46^,^47^,^48^,^49^. The main conclusion from these studies is that great ape communication is intentional and that they act as if taking into account basic mental states of their recipients, such as attention and comprehension. What remains unknown, however, is whether any of their natural communication efforts are also based on taking into account familiarity, i.e. what others know based on shared interactions history.

We addressed this point with a study on bonobos living under semi-natural conditions using a combination of well-established experimental protocols developed for apes (e.g.^38^,^46^,^50^) and human infants^7^. Specifically, we tested whether subjects adjusted signal production to request food from a human partner depending on (a) the recipient’s attention (‘Attentive’ vs. ‘Inattentive’), (b) the success in communicating a goal (‘Fully successful’, ‘Partially successful’, or ‘Unsuccessful’) and (c) the familiarity of a human recipient (‘Familiar’ vs. ‘Unfamiliar’). While the first capacity (‘attention’) appears to be generally available to great apes and other social mammals^34^,^51^, the second one (‘communicative success’) has only been tested in orang-utans and chimpanzees^46^,^47^,^48^, while the third one (‘familiarity’) has only been tested in human children interacting with adults^7^,^52^.

## Predictions

### Recipient attention (a)

If bonobos are able to take others’ attentional state into account, they should adjust their signalling behaviour in accordance with the visual attention of the recipient. This ability has been demonstrated for other great apes in similar experimental protocols and for all great ape species during natural communication^25^,^26^,^28^. If subjects have an understanding of the communicative function of their signals, then they should indifferently use silent or audible signals provided the recipient is visually attentive. If the recipient is not visually attentive, however, subjects should decrease the use of silent gestures and increase, or at least maintain, the rate of audible signals relative to the attentive condition

### Communicative success (b)

If bonobos are able to take their own signalling success into account, they should stop signalling when successful, repeat signals when partially successful (‘Repetition’) and switch to new signals when not successful (‘Elaboration’), as previously shown for chimpanzees and orang-utans^46^,^47^,^48^,^49^.

### Recipient familiarity (c)

If bonobos have some understanding of the fact that they only have a shared interaction history with a familiar recipient, then they should adjust their communicative behaviour accordingly. In the case of a communication failure, subjects should show persistence by repeating previously successful signals (i.e. signals that led to obtaining desirable food in past interactions) when communicating with a familiar recipient. In contrast, when failing to communicate with an unfamiliar recipient (with no shared history of interaction), subjects should elaborate by favouring alternative signals to increase their chances of being understood.

## Materials and Methods

### Ethics statement

The experiment was performed in accordance with the ethical ASAB/ABS Guidelines for the Use of Animals in Research and was conducted in compliance with animal care regulations and applicable national laws (research permit: MIN.RS/SG/004/2009). We received ethical approval from the scientific coordinator and scientific committee of ‘Les Amis des Bonobos’ for this study.

### Study group

We collected data from 10 individuals in three social groups housed at the ‘Lola Ya Bonobo’ sanctuary, DRC, in January 2013. All groups live in large forested enclosures of 8, 10 and 15 ha, respectively, composed of patches of primary rainforest, lakes, swamps, streams, and open grassy areas. During the day individuals can move freely, forage for wild fruits, leaves, and herbaceous vegetation in the forested parts of their enclosures, in addition to three feedings provided by caregivers. The daily feeding routine is to distribute fruits in the morning, a mixture of soya milk (supplemented with milk, maize, honey and nutriments) around midday, and vegetables in the afternoon, approximately 6 kg of fruits and vegetables to each individual, complemented with daily supplemental feeds of seasonal fruits and nuts. Water is freely available from lakes, ponds and streams within their enclosures, with fresh water (with added salt and sugar) provided several times a week. At night, all individuals are kept in dormitories of approximately 75 m^2^, divided in several separable rooms and connected to the outside enclosures by a tunnel. During the study period, group 1 consisted of 25 individuals, group 2 of 16 individuals and group 3 of 11 individuals. All groups included adult, subadult, juvenile and infant males and females.

### Experimental protocol

Twelve subjects were initially selected for this study, either because they produced idiosyncratic signals to request food (*n* = 4; Supplementary Table S1), probably developed through repetitive interactions with a familiar keeper, or because they could be easily tested with regards to their social position in the group. We had to exclude two individuals due to lack of motivation to interact with the human partners or because of a transient lack of interest in the foods provisioned. Consequently, the final analyses were carried out on the performance of 10 individuals (Supplementary Table S2).

All individuals habitually requested food from the keepers with gestures, vocalisations or combinations of both (Supplementary Tables S1 and S3). We first collected a database of signals produced by the subjects to request food from their keepers during daily feedings. For each individual we then selected a random sample of 10 video clips containing food requests from the subjects during these daily feedings with their main keepers, i.e. 100 clips total, from a sample of 192 clips collected during a two-week period (*n* = 10; mean ± SD = 17.45 ± 5.32).

We then classified and ranked the signals by frequency of use. Signals that accounted for at least 80% of a subject’s signalling production were considered as the ‘default’ signals to request food from its keeper, which was often a hand-reach gesture, a general begging gesture of great apes^53^,^54^ and humans (e.g. Supplementary Table S1). We also found that 4 of 10 individuals additionally produced idiosyncratic signals as their personal ‘default’ signals (e.g., hand-clap/rasp grunt or hand-reach/raspberry, Supplementary Table S1 & Videos S1–4).

**Figure 1:**
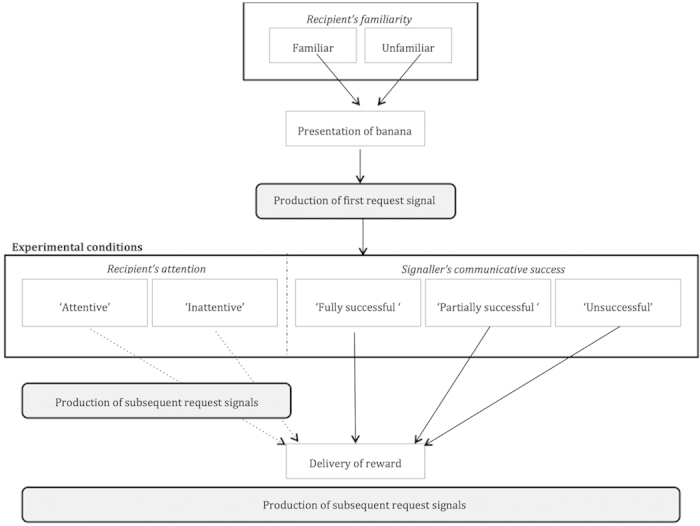
Schematic representation of the experimental protocol with five experimental conditions and two recipients. White boxes represent recipients’ actions and grey boxes represent signallers’ actions. Plain arrows represent an immediate transition to the next box, dotted arrows represent a 60 s delay before the delivery of the reward (a banana in the ‘Attentive’, ‘Inattentive’ and ‘Fully successful’ conditions, half a banana in the ‘Partially successful’ condition, and a corn in the ‘Unsuccessful’ condition). Italic captions represent the variables that are being tested: adjustment of signal production to (**a**) recipient’s attention (‘Attentive’ (1) and ‘Inattentive’ (2)), (**b**) communicative success (‘Fully successful’ (3), ‘Partially successful’ (4), ‘Unsuccessful’ (5)) and (**c**) familiarity of recipient (‘Familiar’, ‘Unfamiliar’).

All experimental trials took place during regular feeding times. To study signalling behaviour, we used banana (desirable) and corn (undesirable) as food incentives for all subjects. The familiar recipients were the main keepers of each group (i.e. three different recipients), who the subjects had been interacting with on a daily basis for years. The unfamiliar recipient was a person recruited from a village nearby the sanctuary whom the subjects had never met before. Each trial began with the human partner approaching the enclosure fence, looking at the subject and calling its name for him/her to approach. When the subject was present and attentive at the fence, the partner showed him or her a banana and, as soon as the subject produced the first request signal, the partner initiated one of five experimental conditions: (1) ‘Attentive’: Recipient facing subject, delivery of banana after 60 s; (2) ‘Inattentive’: Recipient turning his back to subject, delivery of banana after 60 s. In both conditions the banana was always equally visible to the subject (displayed in front or back of recipient); (3) ‘Fully successful’: Immediate delivery of whole banana, (4) ‘Partially successful’: Immediate delivery of half banana; (5) ‘Unsuccessful’: Immediate delivery of corn (hidden in food box during banana presentation) (Fig. 1). Each individual completed a total of 10 trials (one trial per condition with each recipient), one condition per day with each recipient (i.e., two conditions per day). Conditions were counter-balanced across subjects and recipients (Supplementary Table S4).

All trials were recorded on video by EG using a Panasonic full HD digital camcorder (HC-X909) equipped with a directional microphone (Sennheiser MKE 400). EG stood about a 1.5 m from the recipient in order to film both his and the subject’s behaviours. At the beginning of each trial, EG announced the experimental condition. In the ‘Attentive’ and ‘Inattentive’ conditions, a trial began as soon as the subject produced a request signal and ended after a period of 60 s. The subjects’ behaviour was then recorded for another 30 s after the delivery of the food.

All signalling behaviours were recorded during the 60 s pre-delivery period and 30 s post-delivery in the ‘Attentive’ and ‘Inattentive’ conditions and during the 30 s post-delivery period in the ‘Fully successful’, ‘Partially successful’ and ‘Unsuccessful’ conditions. The subjects’ patterns of signalling across the two periods was then classified as (1) ‘Repetition’: number of repetitions of the first request signal in the bout (almost always the ‘default’ signal), (2) ‘Elaboration’: number of new signals in the bout (different signals from the first one). If a signal, other than the first one, was repeated in the bout it was not counted as a ‘Repetition’.

Differences in signalling patterns across conditions and recipient were analysed by assessing all signals produced during the 60 s of the ‘Attentive’ (1) and ‘Inattentive’ (2) conditions (subjects never persisted in signalling following delivery of the food in these two conditions) and the 30 s post-delivery in the ‘Partially successful’ (4) and ‘Unsuccessful’ (5) conditions (subjects never persisted in signalling after delivery of the food in the ‘Fully successful’ (3) condition).

All trials were coded from video by EG, using Filemaker Pro. A second rater, naïve to the hypotheses, but familiar with bonobo communicative signals, recoded 20% of the videos to assess inter-observer reliability. Inter-observer reliability was excellent for both gesture types (K = 0.93) and vocalisations types (K = 0.83).

### Statistical analyses

To investigate whether subjects were able to adjust the signal modality to the attentional state of the recipient (a), we used non-parametric Wilcoxon paired-samples tests to compare production frequencies of silent and audible signals across the ‘Attentive’ and ‘Inattentive’ experimental conditions, pooled across ‘Familiar’ and ‘Unfamiliar’ recipients. We opted for this analysis strategy because we were not primarily interested in differences between recipients, but in the effect of the recipients’ attentional state on signal production.

In a second analysis, we used generalized linear mixed models^55^ with a binomial error and logit link function to test for differences in signalling patterns (i.e. ‘Repetition’ and ‘Elaboration’) across (b) experimental conditions (‘Attentive’, ‘Inattentive’, ‘Partially successful’ and ‘Unsuccessful’) and (c) recipient familiarity (‘Familiar’ and ‘Unfamiliar’). In two separate models, we modelled the proportion of signals that constituted ‘Repetition’ and ‘Elaboration’, respectively, from a database consisting of all signals produced in a given bout. Predictor variables in both models were experimental condition and recipient familiarity. Subject ID was fitted as random factor. Variance inflation factors were all <1.1, indicating that collinearity was not a problem. We assessed model stability by calculating Cook’s distance^56^, which indicated one influential individual in the ‘Repetition’ model. Reanalysing the data excluding this individual did not change our conclusions derived from the model and we present results with this subject included. We used likelihood ratio tests (LRT^57^) to assess significance of full models versus null models (containing only intercept and random effect) as well as for our categorical predictor variables.

The overall analysis strategy is summarised in Table 1. Models were fitted in R 3.1.1^58^ with the function glmer in the lme4 package^59^. Variance inflation factors were calculated with the vif function in the car package^60^, Wilcoxon tests with exact p-values from the exactRankTests package^61^, and Cook’s distances with the influence. ME package^56^.

## Results

### Recipient attention (a)

**Figure 2:**
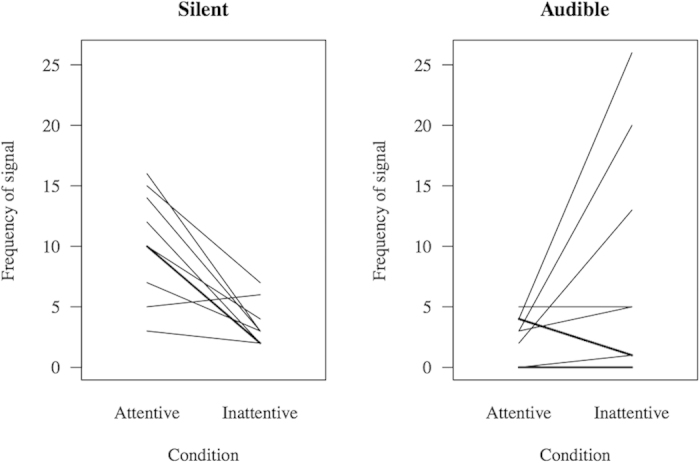
Differences in signal modality according to recipient attentional state. Each line represents one subject, except thick lines, which indicate that two subjects produced the same number of signals in both conditions. Numbers were pooled across familiar and unfamiliar recipients for each subject.

As predicted, we found that subjects adjusted signal modality to the attentional state of their recipient by using fewer silent signals in the ‘Inattentive’ compared to the ‘Attentive’ condition (V = 1.5, p = 0.0059, N = 10 with 1 tie; median ‘Inattentive’ = 3.00, inter-quartile range: 2.00–3.75; median ‘Attentive’ = 10.00, inter-quartile range: 7.75–13.50, Fig. 2). For audible signals, we found no difference according to the attentional state of the recipient (V = 7, p = 0.2969, N = 10 with 3 ties; median ‘Inattentive’ = 3.00, inter-quartile range: 1.00–11.00; median ‘Attentive’ = 3.00, inter-quartile range: 0.50–4.0, Fig. 2), although the observed pattern went in the expected direction, with only two subjects presenting lower frequencies of audible signals in the ‘Inattentive’ condition.

**Figure 3:**
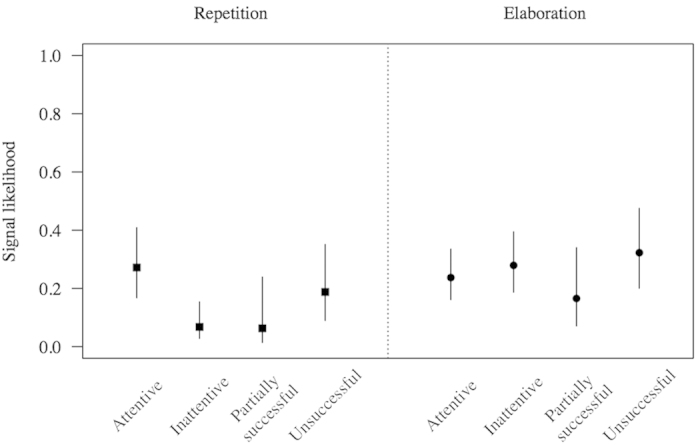
Results of the GLMMs testing differences in signal repetition and elaboration according to experimental condition. Shown are model estimates with associated standard errors.

Although it was not the focus of this analysis, the result of the models (see below) indicated that the subjects used fewer repetitions with an inattentive recipient (estimate = −1.64, SE = 0.46, z = −3.59, p = 0.0003) than with an attentive one (see Fig. 3). However, we found no difference in elaboration with regard to the attentional state of the recipient (estimate = 0.22, SE = 0.31, z = 0.70, p = 0.4867).

### Communicative success (b)

In the ‘Fully successful’ condition, no subject made any further attempts to communicate with the recipient after the delivery of the banana. In the ‘Partially successful’ condition, all 10 subjects immediately ate half the banana and only 3 out of 10 continued to signal afterwards (two only did so with the unfamiliar recipient). In the ‘Unsuccessful’ condition, half the subjects ate the corn but only after subsequent attempts to obtain the banana had failed.

Both full models differed from their respective null models (Repetition: χ^2^ = 32.7, df = 4, p = 0.0000, Elaboration: χ^2^ = 8.3, df = 4, p = 0.0817, Tables 2 and 3). We found that bonobos tended to persist more by repeating the first request signal (estimate = 1.24, SE = 0.84, z = 1.48, p = 0.1394, Fig. 3) and to elaborate more by producing new signals (estimate = 0.88, SE = 0.55, z = 1.58, p = 0.1134, Fig. 3) in the ‘Unsuccessful’ compared to the ‘Partially successful’ condition. Although statistically not significant, the directions of these results were consistent with our predictions.

### Recipient familiarity (c)

**Figure 4:**
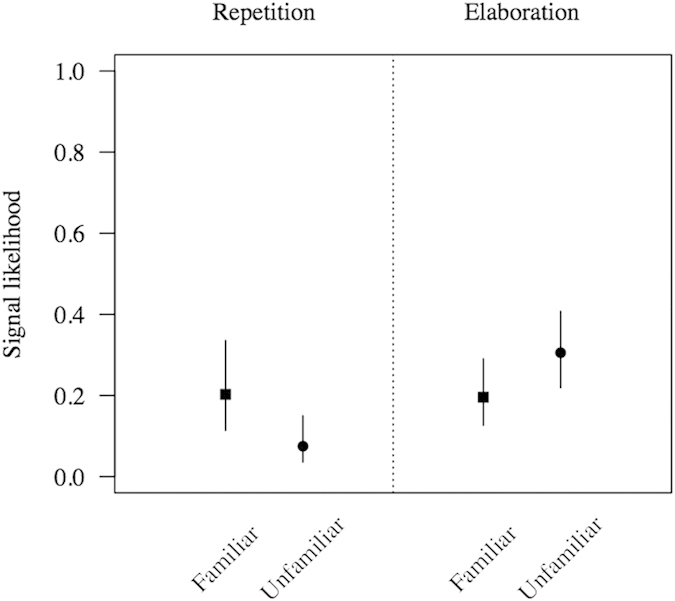
Results of the GLMMs testing differences in signal repetition and elaboration according to recipient familiarity. Shown are model estimates with associated standard errors.

As predicted, bonobos used fewer repetitions with the unfamiliar recipient compared to the familiar ones (estimate = −1.15, SE = 0.34, z = −3.36, p = 0.0008, Fig. 4), but at the same time elaborated more by using more new signals with the unfamiliar than the familiar ones (estimate = 0.59, SE = 0.27, z = 2.18, p = 0.0290, Fig. 4).

## Discussion

In this study, we found that bonobos communicated with a range of signals to obtain desirable food from human recipients in goal-directed and strategic ways. With our first analysis, we were able to replicate findings of earlier studies (e.g.^33^,^34^,^35^,^37^,^38^,^39^,^50^) by showing that bonobos take into account the attentional states of their recipients during communication. With our second analysis, we were able to replicate findings in orang-utans and chimpanzees^46^,^47^,^48^,^49^, demonstrating that bonobos can adjust and refine communication signals depending on the degree of success of previous communication efforts. If successful in obtaining the banana, subjects never continued to signal, but if unsuccessful they tended to elaborate their communicative efforts by switching to new signals, suggesting an apparent attempt to rectify an unsuccessful communication event and misunderstanding. With our third analysis, we showed that, similarly to human infants interacting with adults^7^,^52^ and language-trained bonobos interacting with their caretakers^14^,^15^, subjects adapted their signal production according to whether or not they knew the recipient. In particular, they used more repetitions with a familiar recipient but elaborated more, by using new signals with an unfamiliar one.

Our results are consistent with the hypothesis that bonobos take into account recipient familiarity, and possibly consider the knowledge they share with recipients that has been acquired jointly during their common history of interactions. Presumably, subjects have learnt, over the course of previous interactions, which signals are likely to persuade the familiar human recipient to deliver a food reward and they seemingly quickly understand that these signals are not necessarily equally effective when interacting with an unfamiliar recipient with whom they have no shared history. Whether this is the result of mere operant conditioning or reasoning cannot be determined here. Operant conditioning theory predicts that a subject learns to associate a previously neutral behaviour (e.g. producing an idiosyncratic gesture) to human recipients with a positive outcome (food). One version of this type of learning has been referred to as ‘conditional discrimination’, i.e. a discrimination between two stimuli in which the reinforcement for responding to one stimulus (food) depends on the presence of another stimulus (human partner)^62^. In our opinion, these results do not support such an interpretation because in conditional discrimination subjects should transfer the initially trained association to any human recipient, regardless of familiarity. The alternative is that signalling behaviour would have had to be learned by conditioning with each recipient separately. However, in our study the unfamiliar recipient was unknown to the subjects and yet we found that signalling behaviour was influenced by this variable in ways that suggest that recipients were aware of the differences. Subjects reacted to the unfamiliar human by changing signals more often than to a familiar one from the first exposure and without training, indicating that request signals are not just blindly acquired and delivered, but deployed strategically by taking the targeted recipient into account.

We find it thus more likely to assume that great apes can partially discriminate between private and shared knowledge when communicating with a partner, something that has recently been suggested for chimpanzee alarm calls^30^. In our current study, this ability has revealed itself without any specific training but while using idiosyncratic signals and other usual food request signals that are part of the bonobos’ natural signalling repertoire.

Familiarity judgements are an important component for the mental capacity to form a mutual ‘common ground’, a key capacity in human social interactions^6^. To this end, humans routinely take the knowledge shared with their social partners into account, both in linguistic conversations^63^,^64^ as well as during joint attention and joint actions^65^. ‘Common ground’ is a hallmark of human cooperative behaviour and communication and, according to some accounts, an exclusive capacity as it is based on the ability to attribute mental states to others^64^,^66^,^67^. For example, if two interlocutors have previously shared an experience relating to an object, they will both know that they can make relevant inferences about this object even in its absence, but only with this partner^8^,^68^, an ability that appears in human infants from 12 months of age^65^.

Although our results appear to be consistent with such an interpretation, simpler mechanisms may have been at work. For example, it could be argued that differences in familiarity generate differences in emotional states, which may affect communication behaviour. However, our study is unable to describe in more detail the cognitive abilities deployed by the bonobos to discriminate between familiar and unfamiliar recipients. Whatever the underlying mechanism, the fact remains that differences in partner familiarity caused differences in signalling behaviour that was structured in a way that suggested subjects took their partners’ knowledge into account. In future research it would be necessary to let subjects interact with a range of familiar and unfamiliar recipients in a matched design (i.e. some recipients being familiar to some subjects but unfamiliar to others). In our case, this would have required 20 human experimenters, something that unfortunately was impractical within the constraints of the sanctuary setting. Instead we opted for a previously established protocol with human children^7^, which enabled us to use the main bonobo keeper for a given enclosure as the ‘Familiar’ recipient and one unfamiliar human partner as the ‘Unfamiliar’ recipient for all individuals, allowing comparisons to the human infant study^7^. Further research is needed to investigate whether experimentally created shared knowledge between a subject and a human partner (e.g. training of novel arbitrary signals with different social partners) has any measurable behavioural consequences and indications of shared knowledge and understanding.

One limitation of the present and similar types of studies is that the experimental design is based on testing interactions with human partners. Obviously, this is very different from natural situations in which individuals are communicating with each other and as part of differentiated relationships. It could therefore be argued that our experimental design is based on a situation that rarely occurs in the wild, i.e. an individual communicating to an unfamiliar partner to obtain food. One interesting exception is the arrival of an immigrant in an established group, a situation that naturally occurs in wild chimpanzees and bonobos. Do immigrants progressively adapt their communication signals on the basis of shared history with the different group members, as recently suggested for the food calls of captive chimpanzees^69^ Documenting the communication behaviour of newly arriving individuals throughout the immigration phase would generate relevant data to study the communicative interactions between individuals of different familiarity. Here, our prediction is that individuals, who are unsuccessful in communicating their goal, should show more persistence (more repetitions) when interacting with already familiar individuals compared to still unfamiliar individuals, to whom they should show more signs of elaboration.

## Conclusion

Parts of the human psychological architecture are shared with the great apes, in particular the ability to flexibly communicate by taking into account the visual attentive state of the recipient and one’s own communicative success. Our study further suggests that bonobos also possess an understanding of the shared history of interaction. What remains unknown, however, is the exact nature of the underlying mechanism responsible for the distinction between familiar and unfamiliar recipients and to what degree mental state attribution is involved.

## Additional Information

**How to cite this article**: Genty, E. *et al.* Bonobos modify communication signals according to recipient familiarity. *Sci. Rep.* **5**, 16442; doi: 10.1038/srep16442 (2015).

## Supplementary Material

Supplementary Video S1

Supplementary Video S2

Supplementary Video S3

Supplementary Video S4

Supplementary Information

## Figures and Tables

**Table 1:** Summary of statistical tests.

Aim	Signal variables	Predictor variable	Test
a	Silent and Audible	Familiarity	Wilcoxon
b	Repetition and Elaboration	Condition	GLMM
c	Repetition and Elaboration	Familiarity	GLMM

**Table 2:** Results of the GLMM testing differences in Repetition of signals.

	estimate	se	z	p
Intercept	−0.41	0.33	−1.25	0.2110
Experimental condition^a^				
Inattentive	−1.64	0.46	−3.59	0.0003
Partially successful	−1.72	0.78	−2.20	0.0278
Unsuccessful	−0.48	0.42	−1.14	0.2546
Recipient familiarity^b^				
Unfamilar	−1.15	0.34	−3.35	0.0008

Note that reference levels for both predictor variables (‘Attentive’ for experimental condition and ‘Familiar’ for recipient familiarity) are included in the intercept.

^a^LRT: χ^2^ = 18.5, df = 3, 0.0003.

^b^LRT: χ^2^ = 12.0, df = 1, p = 0.0005.

**Table 3:** Results of the GLMM testing differences in Elaboration of signals.

	estimate	se	z	p
Intercept	−1.46	0.28	−5.20	0.0000
Experimental condition^a^				
Inattentive	0.22	0.31	0.70	0.4867
Partially successful	−0.45	0.51	−0.88	0.3806
Unsuccessful	0.43	0.37	1.15	0.2509
Recipient familiarity^b^				
Unfamilar	0.59	0.27	2.18	0.0290

Note that reference levels for both predictor variables (‘Attentive’ for experimental condition and ‘Familiar’ for recipient familiarity) are included in the intercept.

^a^LRT: χ^2^ = 3.2, df = 3, 0.3634.

^b^LRT: χ^2^ = 4.9, df = 1, p = 0.0273.
