# Supplementary material for: Bonobos modify communication signals according to recipient familiarity
Source: Sci Rep. 2015 Nov 10;5:16442. doi: 10.1038/srep16442 (PMC4639733; doi:10.1038/srep16442)
Supplement: Supplementary Information [file srep16442-s5.pdf]

Bonobos modify communication signals according to recipient familiarity

Emilie Genty[a,*], Christof Neumann[a], and Klaus Zuberbühler[a, b]

Authors affiliation: [a] Department of Comparative Cognition, Institute of Biology, University of Neuchâtel, rue Emile Argand 11, 2000 Neuchâtel, Switzerland. [b] School of Psychology and Neuroscience, University of St Andrews, St Andrews, KY16 9JP, Scotland (UK)

***Corresponding author:** Emilie Genty, Department of Comparative Cognition, Institute of Biology, University of Neuchâtel, rue Emile Argand 11, 2000 Neuchâtel, Switzerland, +41 (0) 32 718 31 15, emilie.genty@unine.ch.

Supplementary information

Supplementary tables

Table S1. Default signals by subjects to request food. Default signaling is defined as the most common request signals accounting for at least 80% of the signals produced during *n* = 10 request episodes. Frequency of use is represented by percentage in brackets. Bold caption represent idiosyncratic signals.

Subject	Default signals
Semendwa	**Arm up reach while standing bipedally** (100%)*
Lisala	**Hand-reach up + raspberry while standing bipedally** (70%)*; Hand-reach up (20%)
Kikwit	Hand-reach front (60%); Hand-reach up (20%)
Dilolo	**Clap hands + Rasp grunt** (50%)*; Hand-reach up (30%)
Maya	Arm raise (40%); Hand-reach up (20%); Head bob (20%)
Bisengo	Hand-reach up (30%), Hand-reach front (30%); Arm raise (10%), Hand shake (10%)
Waka	Arm raise while standing bipedally (50%); **Arms raise while standing bipedally (30%)***
Isiro	Hand-reach front (60%); Hand-reach up (40%)
Likasi	Hand-reach up (60%); Hand-reach front (40%)
Shibo	Arm raise (50%); Hand-reach up (20%), Stomp (20%)

* Supplementary Movies S1, S2, S3 and S4.

Table S2: List and description of subjects

Group	Subject	Sex	Age class	Social status
1	Semendwa	Female	Adult	High
1	Lisala	Female	Subadult	High
1	Kikwit	Male	Adult	Intermediate
1	Dilolo	Male	Subadult	Intermediate
2	Maya	Female	Adult	High
2	Bisengo	Male	Subadult	Intermediate
2	Waka	Female	Subadult	Low
3	Isiro	Female	Adult	High
3	Likasi	Female	Subadult	Intermediate
3	Shibo	Male	Subadult	Low

Table S3: List and definition of recorded signals to request food during experiment.

**Silent gestures**	
Arm raise	Raising one arm above head
Arms raise	Raising both arms above head
Arm reach up	Raising one arm laterally on side of body
Arm swing	Swinging arm back and forth on side, either once or repetitively
Hand-reach front	Stretching arm and hand toward another individual, arm is stretched in front of self
Hand-reach up	Stretching arm and hand toward another individual, arm is up above head level
Hand shake	Shaking hand loosely from wrist joint
Fingers flex	Stretching hand out toward another individual with rapid alternation of stretching and bending movements of fingers
Head bob	Bobbing head up and down
**Auditory gestures**	
Body beat	Drumming body part with palm of hands
Clap hands	Tapping both palms of hands against each other
Punch object	Punching singly object or ground with closed hand
Stomp	Stamping the ground forcefully with sole of foot
Throw object	Throwing object at another individual
**Vocalisations**	
	Rasp grunt, Raspberry, Peep, Moan, Hoot, Bark, Mix series

Table S4: Order of presentation of experimental conditions with 'Familiar' and 'Unfamiliar' recipients for all subjects.

Subjects	Familiar recipient					Unfamiliar recipient				
Semendwa	1	5	2	4	3	3	5	1	2	4
Dilolo	1	2	3	4	5	3	4	2	1	5
Kikwit	3	4	2	1	5	2	3	4	1	5
Lisala	4	1	2	3	5	5	4	2	3	1
Bisengo	1	5	2	4	3	3	5	1	2	4
Maya	3	4	1	2	5	2	3	4	1	5
Waka	3	2	1	4	5	4	1	2	3	5
Likasi	4	3	2	1	5	1	4	3	2	5
Isiro	1	5	4	3	2	5	1	2	4	3
Shibo	2	5	1	4	3	4	5	1	3	2

(1): 'Attentive'. (2): 'Inattentive', (3): Fully successful'; (4): 'Partially successful'; (5): 'Unsuccessful'.

Supplementary videos

Video S1: Illustration of the idiosyncratic signal produced by the adult female Semendwa, consisting of an arm up while standing bipedally.

Video S2: Illustration of the idiosyncratic signal combination produced by the subadult male Dilolo, consisting of clapping hands combined with a rasp grunt

Video S3: Illustration of the idiosyncratic signal combination produced by the adult female Lisala, consisting of a hand-reach up combined with a raspberry sound while standing bipedally.

Video S4: Illustration of the idiosyncratic signal produced by the subadult female Waka, consisting of both arms raised while standing bipedally.
